# Supplementary material for: Towards inclusive risk-informed infrastructure development in expanding cities
Source: Commun Eng. 2025 Sep 2;4:161. doi: 10.1038/s44172-025-00494-3 (PMC12405530; doi:10.1038/s44172-025-00494-3)
Supplement: Supplementary file 2 — Supplementary Information [file 44172_2025_494_MOESM2_ESM.pdf]

Supplementary Information for:

"Towards inclusive risk-informed infrastructure development in expanding cities"

Fabrizio Nocera[1*], Yahya Gamal[2,3*], Chenbo Wang[4], and Gemma Cremen[5]

[1] *Postdoctoral Research Fellow, Department of Civil, Environmental and Geomatic Engineering, University College London, London, UK. E-mail:* f.nocera@ucl.ac.uk

[2] *Research Associate, Urban Big Data Centre, University of Glasgow, Glasgow, UK. E-mail:* yahya.gamalaldin@glasgow.ac.uk

[3] *Research Assistant, Department of Geography, King's College London, London, UK.*

[4] *PhD Student, Department of Civil, Environmental and Geomatic Engineering; University College London, London, UK. E-mail:* chenbo.wang@ucl.ac.uk

[5] *Lecturer, Department of Civil, Environmental and Geomatic Engineering; University College London, London, UK. E-mail:* g.cremen@ucl.ac.uk

**Equal first authors*

Supplementary Note 1

We explore the variation of gentrification (i.e., the number of expected triggered relocations, $E(\varepsilon)$) with infrastructure performance (in terms of Z) for the 20 road expansion possibilities with the highest Z that comply with all constraints of the optimisation except (possibly) $E(\varepsilon) \leq \varepsilon_{\mathrm{T}}$ (see Supplementary Figure 1). These results reveal an unclear (complicated) relationship between Z and ε; it is not always the case that better infrastructure performance entails an additional gentrification cost. This is because larger values of Z that enhance the connectivity (utility) of residential unit r lead to increases in both a buyer's willingness to pay ($\mathrm{WTP}_{r,b}$) and the prices set by the seller ($P_{r,s}$), and it is the trade-off between the two that ultimately determine $\mathrm{E}(\varepsilon)$. This exploratory analysis underlines the importance of tracking both Z and ε for informed, responsible decision making.

In each case, all $E(\varepsilon)$ values are associated with low-income households. This can be explained by the general similarity of $\mathrm{WTP}_{r,b}$ and $P_{r,s}$ values associated with these households (more details to follow). It also underlines the strong spatial correlation that exists between lack of income and level of exposure to flooding in Tomorrowville[1]. The infrastructure performance optimisation process pays specific attention to increasing the connectivity of (and therefore driving price increases in) flood-prone areas, given its risk mitigation goal expressed through Z_2 (see Equation (8)). The pro-poor nature of the optimisation process adopted is another reason why infrastructure development is predominantly focused in low-income areas.

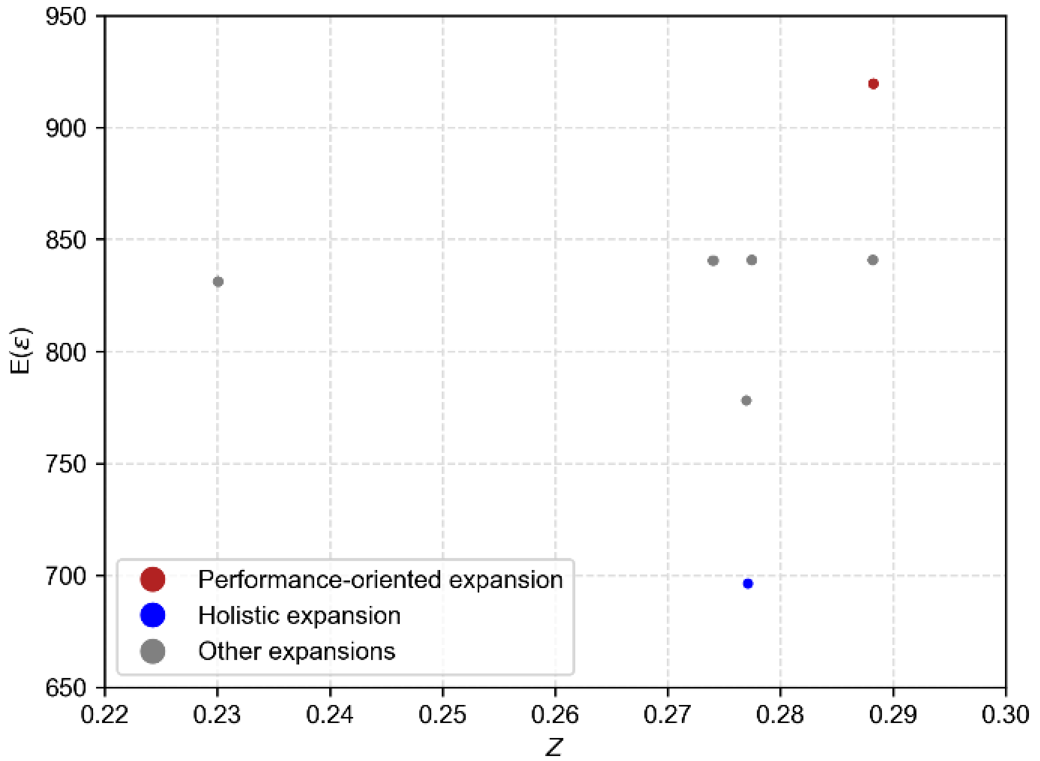

Supplementary Figure 1. Variation in expected gentrification, $E(\varepsilon)$, with infrastructure performance, Z, across the 20 infrastructure development proposals with the highest Z.

Supplementary Note 2

We introduce a further set of experiments aimed at understanding the relationship between prices set by the seller, $P_{r,s}$, and the number of resulting triggered relocations (i.e., the number of occurrences of the price exceeding the willingness to pay of the buyer $P_{r,s} > \mathrm{WTP}_{r,b}$), disaggregated by income. We increase all $P_{r,s}$ values obtained for the baseline (existing) Tomorrowville road infrastructure by a given percentage and re-calculate $E(\varepsilon)$, keeping all other parameters the same. The results (see Supplementary Figure 2) reveal that for price increases up to 10%: (1) there are no expected triggered relocations of high-income households, whose available budget is sufficient to mean that their willingness to pay still exceeds the prices set; (2) expected triggered relocations for middle-income households start when the price increases by approximately 6%; and (3) expected triggered relocations for low-income households grow exponentially with percent increase in price, indicating that these households have willingness to pay values that are very close to the original prices set by the seller. In summary, these experiments confirm that low-income households would remain particularly susceptible to triggered relocations, even if rental prices increased somewhat.

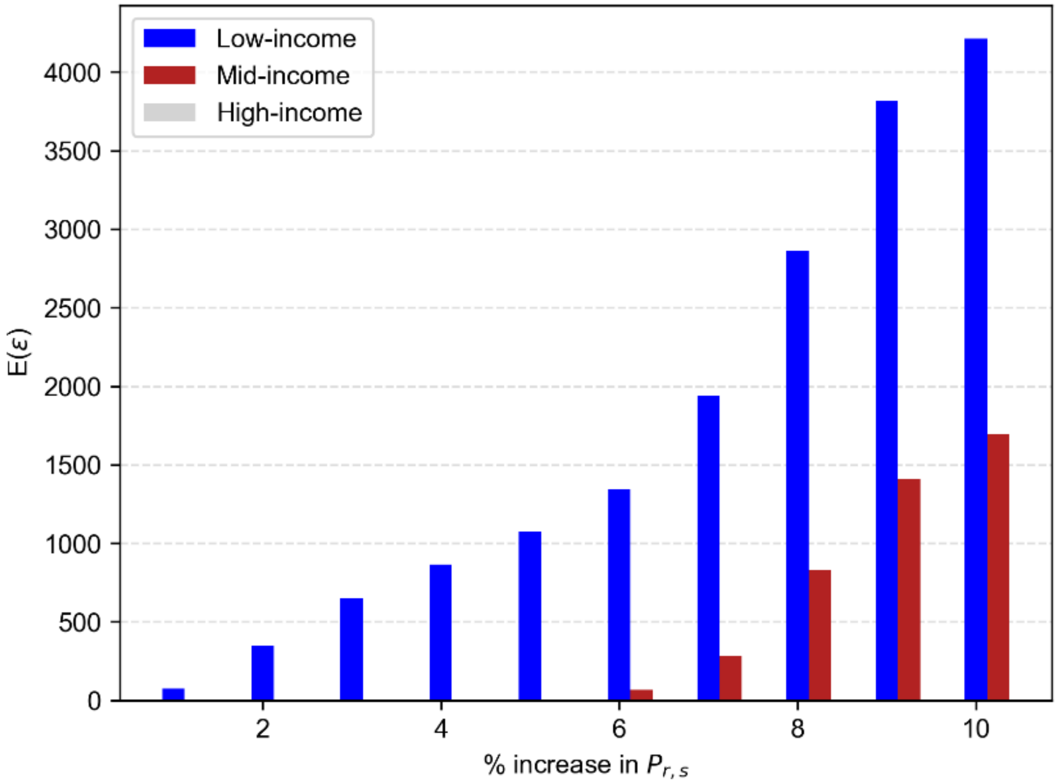

Supplementary Figure 2. Variation in expected gentrification, $E(\varepsilon)$, if there are one to 10 percent increases in the prices set by the seller, $P_{r,s}$, considering the existing Tomorrovwille road infrastructure.

Supplementary References

1. Agrawal, H., Wang, C., Cremen, G., & McCloskey, J. (2024). A geophysics-informed pro-poor approach to earthquake risk management. *Natural Hazards*, 1-19.
